# Using Digital Technology to Build COVID-19 Vaccine Confidence: A Qualitative Study among Latinx Parents of Children Aged 5–11 in Under-Resourced Communities across Los Angeles County

**DOI:** 10.3390/vaccines11061042

**Published:** 2023-05-30

**Authors:** Michael Panameno, Luisa R. Blanco, Ann Marie Hernandez, Renato Escobar, Brittney Zendejas, Susana Rafaela, Yelba M. Castellon-Lopez

**Affiliations:** 1 David Geffen School of Medicine, University of California, Los Angeles, CA 90069, USA; mpanameno@mednet.ucla.edu (M.P.); annmariehernandez@mednet.ucla.edu (A.M.H.); 2 College of Medicine, Charles R. Drew University of Medicine and Science, Los Angeles, CA 90069, USA; 3 Pepperdine School of Public Policy, Pepperdine University, Malibu, CA 90069, USA; luisa.blancoraynal@pepperdine.edu; 4 Department of Family Medicine, David Geffen School of Medicine, University of California, Los Angeles, CA 90069, USA; renny760@g.ucla.edu (R.E.); brittneyz@g.ucla.edu (B.Z.); susyrafaela03@g.ucla.edu (S.R.); 5 Department of Biomedical Sciences, Cedars-Sinai Medical Center, Samuel Oschin Comprehensive Cancer Institute, Cancer Research Center for Health Equity, 700 N San Vicente Blvd, PDC Green, G500, West Hollywood, CA 90069, USA

**Keywords:** COVID-19, vaccination, Latinx children, vaccine confidence

## Abstract

**Background**: Latinx school-aged children are more than twice as likely to be infected with and die from COVID-19 as non-Latinx White children in Los Angeles. Although COVID-19 vaccination has the potential to mitigate health disparities exacerbated by the pandemic, vaccination uptake among Latinx children remains limited. MiVacunaLA (MVLA) is a mobile-phone-delivered digital intervention that improved vaccination rates in 12- to 17-year-old Latinx children and parental intention to vaccinate 2- to 11-year-old children. Since piloting MVLA, the COVID-19 vaccine became available to children aged 5–11. We sought to understand parental experiences with the MVLA intervention and their attitudes and beliefs about vaccinating their young children to improve vaccination confidence in the Latinx community. **Methods:** We conducted six virtual focus groups with 47 parents/caregivers of children aged 5–11 who participated in the MVLA intervention. We used standard qualitative content analysis methods and rigid and accelerated data reduction to identify and analyze major themes discussed in the sessions. **Results:** Each salient theme from our focus groups was mapped to one of the 5Cs constructs. The themes included the parents’ need for more contemplation about vaccinating their children than about vaccinating themselves; the parents’ need for trusted sources of vaccine information; the parents’ motivations to vaccinate their children against COVID-19; parental concern about short- and long-term effects of the vaccine in children; digital technology and videos as useful engagement tools; and age and health stratification as an approach to parental vaccination decision-making. **Conclusions:** The results of this study clarify the key factors that influence the decision of Latinx parents and caregivers to vaccinate their children against COVID-19. Our findings can inform efforts to increase COVID-19 vaccination rates among children in underserved Latinx communities, especially regarding the use of digital technologies for promoting vaccine confidence.

## 1. Background

The COVID-19 vaccine has proven to be a safe and effective approach to prevent severe COVID-19 infections, reducing transmission, and potentially preventing long-term sequelae [1,2]. Multiple seminal studies on COVID-19 vaccination affirm the high efficacy in preventing hospitalization, and death, along with robust immune responses and real-world effectiveness [1,2,3]. These findings collectively support the crucial role of COVID-19 vaccination in controlling the virus’s spread and mitigating its impact on public health and vulnerable communities. However, despite the benefits of COVID-19 vaccination, several studies have demonstrated low confidence in COVID-19 vaccines, especially among racially and ethnically minoritized groups [4,5,6,7,8,9]. A scoping review of vaccine hesitancy in high-income countries by Aw et al. found that in 46 studies (nearly half of the studies included in their review), the rate of vaccine hesitancy was 30% or greater [3,10]. Given such high rates of vaccine hesitancy in the general population, many public health experts, researchers, and policymakers have highlighted the need to build trust, address structural and access barriers, and dedicate resources to promote vaccine confidence among vulnerable populations, including children [4,5,9,10,11,12,13,14,15,16].

On 29 October 2021, the U.S. Food and Drug Administration (FDA) authorized the Pfizer-BioNtech and Moderna COVID-19 (SARS-CoV-2) vaccines for emergency use in children 5–11 years of age [17]. In Latinx children, vaccine uptake remains low. A recent national survey by the Kaiser Family Foundation demonstrated that 35% of parents said that they would “definitely not” vaccinate their 5- to 11-year-old children [18]. In Los Angeles County, where Latinx residents make up the largest ethnic group, only 26.9% of Latinx children between the ages of 5 and 11 had received at least one dose of the COVID-19 vaccine as of January 2023 [19]. Latinx parents who do vaccinate their children against COVID-19 have unique concerns that influence their decision-making, including finding a trustworthy vaccination site and navigating antivaccine sentiments from English-language informational sources [14,15,20,21]. Increasing COVID-19 vaccination rates among children and ensuring equitable access to vaccines is an important strategy to mitigate the impact of the pandemic and thereby promote health equity, especially among those communities that have been disproportionately impacted by the pandemic [4,5,13]. Latino families face a higher prevalence of structural and socioeconomic factors that increase the risk of COVID-19. These factors include over-representation in essential worker roles and living in crowded and multigenerational housing [22,23,24,25]. These risks are compounded by additional structural barriers such as decreased access to health insurance and healthcare services [26,27]. A recent study found that Latino children were also less likely to return to in-person learning during the pandemic due to parental fears about COVID-19, resulting in a downstream impact on educational outcomes for school-aged children [28].

Historically, local and state-wide school vaccination requirements have played an important role in reducing the risk of vaccine-preventable diseases. Given this precedent, the Los Angeles Unified School District initially announced a COVID-19 vaccination mandate for its students, which was later delayed and ultimately struck down by a court ruling [29]. In search of innovative approaches to increasing COVID-19 vaccination rates among children and teens, a local children’s hospital partnered with the Los Angeles County Department of Public Health to sponsor the “VaxUp Challenge,” which called for creative solutions that addressed challenges and overcame barriers in vaccinating children against COVID-19 [30]. We present qualitative focus group data, which were collected as part of the VaxUp Innovation Challenge and used to tailor a mobile-phone-delivered educational intervention called MiVacunaLA (MVLA) [31].

We collected and analyzed qualitative data from Latinx parents and caregivers who participated in MVLA, an intervention developed to increase COVID-19 vaccine uptake in underserved immigrant communities [32]. The MVLA intervention was created through a community-partnered approach to offer a digital, mobile-phone-delivered curriculum aiming to empower Latinx parents with high-yield educational information about the COVID-19 vaccine in children. The intervention consists of short videos and informational material about the COVID-19 vaccine in children, which participants accessed on an online platform via text message. MVLA participants received two text messages per week for 4 weeks, one of which invited them to watch a video and the other to read information related to the specific material covered that week. Information shared with the community was short and concise: the videos were in the 2- to 3-min range and the informational blurbs were in the 400- to 600-word range. All information was available in Spanish and English, and the material was delivered in the language that each participant chose at the beginning of the study. Reminders to complete intervention activities were also sent via email as an additional channel to motivate participants to complete intervention activities.

For this qualitative study, we conducted focus groups with parents who participated in MVLA. We intentionally selected both parents who completed the intervention and those who did not to discuss their views on vaccinating their children and their experience learning about the COVID-19 vaccine through the MVLA intervention. We sampled parents who had children in the 5–11 age group to tailor future iterations of the intervention to children as vaccines were newly authorized for those under 11 years of age. The purpose of our study was to build an understanding of Latinx parental perspectives and decision-making factors regarding COVID-19 vaccination for their children. We used the 5Cs framework to develop open-ended questions and provide a guiding structure for themes that arose in the focus groups [33]. The findings of this qualitative study were used to inform the design of an improved version of the intervention, MVLA 2.0. The findings can further be used to inform public health efforts to increase COVID-19 vaccination efforts among Latinx immigrant parents residing in underserved communities.

## 2. Methods

We used purposeful sampling of parents who reported having a child in the 5- to 11-year age group from those who previously enrolled in the MVLA intervention and consented to be contacted for future studies. We randomly recruited participants who enrolled and completed the MVLA intervention and those who enrolled and did not complete MVLA using a 1:1 sampling ratio. Focus group recruitment was completed in March and April of 2022, shortly after the FDA emergency authorization for the mRNA COVID-19 vaccine for children 5–11 years of age [17]. We used the 5C framework, a validated tool to assess psychological antecedents of vaccination, to create focus group questions in the following domains of vaccination perspectives: confidence (attitude), complacency (perceived personal health status and invulnerability), constraints (self-control), calculation (preference for deliberation), and collective responsibility (communal orientation) [33]. We also collected qualitative data about participants’ experiences with the MVLA intervention to better understand the facilitators and challenges of using digital educational interventions in an under-resourced setting.

We conducted six virtual focus groups (three with participants who completed MVLA and three with those who did not) via Zoom that lasted up to 60 min and were conducted in Spanish by native speakers and trained members of the research team. We included 5–10 participants per group, and we organized focus groups by study completion status to allow for comparisons between those who completed the intervention and those who did not. The Institutional Review Board approved this study, and we reported our findings following the Standards for Reporting Qualitative Research [33]. Table 1 presents selected questions from our focus group guide.

### 2.1. Data Collection

One trained, bilingual member of the research team (MP, LB, and YCL) conducted each focus group while a second researcher (RE) took notes and monitored the virtual platform. Sessions were recorded, transcribed, and translated into English, and native-Spanish-speaking facilitators verified the translations. We asked participants to remove personal identifiers from their Zoom settings and turn off their videos to protect their confidentiality during the recorded sessions. The trained facilitator used a focus group discussion guide with open-ended questions and prompts to maintain fidelity. We used the 5Cs framework to develop our focus group questions that covered domains including participant attitudes, perceptions, and preferences regarding the COVID-19 vaccine for children and their experiences with the MVLA digital intervention (see Table 1). We asked participants to share their perspectives as a parent or caregiver and those they had heard or discussed in their community. We collected data iteratively until we achieved theoretical saturation of emergent themes (when we noted the repetition of answers for a given topic or question) and subsequently performed a constant comparative analysis of the emergent themes. We gave each participant a $40 gift card for participating in this qualitative study.

### 2.2. Analysis

We analyzed transcripts using Dedoose, a web-based platform for analyzing qualitative data. Using a constructivist grounded theory approach, two experienced coders (MP and YCL) reviewed the transcripts and field notes from three sessions to develop a preliminary codebook, which the team then tested and amended. Each transcript thereafter was coded by a pair of trained coders (MP, BZ, SR, and AH) who reached a consensus on the definitions, coding approach, and thematic evolution through weekly meetings to resolve any incongruency throughout the analysis. We achieved triangulation through iterative discussions among all facilitators and research team members and used rigid and accelerated data reduction to reduce the codes to the final themes and subthemes [34].

## 3. Results

### 3.1. Participants

We screened 137 individuals for this study, and 47 parents ultimately participated. We conducted six focus groups with 5–10 people each. The participants had a mean age of 41 years (SD 7.9) and an average of two children in the household. Most participants reported speaking Spanish as their primary language (89.4%) and were born outside of the U.S. (80.9%). A majority were identified as Mexican/Mexican American (66%). Close to half of the participants reported their highest educational attainment as some high school or less (44.7%). Over half of the participants had health insurance (57.5%), and 80% had an annual household income under $25,000 (US dollars). Table 2 reports the participant demographics.

### 3.2. Themes

Each main theme and subtheme that emerged from the data regarding building vaccine confidence was mapped to one of the 5Cs constructs. Here, we present the themes and relevant subthemes further illustrated by participant quotations. Table 3 summarizes our study themes and subthemes and their corresponding 5Cs domains.

#### 3.2.1. Calculation

*a. Vaccinating children against COVID-19 requires more contemplation than vaccinating adults.* A salient theme that arose regarding parental decision-making about COVID-19 vaccination was that deciding to vaccinate children against COVID-19 requires additional contemplation compared with the decision to vaccinate adults. At the same time, parents expressed a need to be informed by trusted sources of information. Participants identified social networks as potential sources of conflicting information. Although their decision to vaccinate their children was affected by COVID-19 vaccination information shared within their social networks, these networks were sometimes sources of misinformation. Some participants expressed a need to continue contemplating vaccination due to their awareness of misinformation and conflicting information. Among the myths described were vaccines causing autism and the COVID-19 vaccine causing long-term cardiac side effects, harming the future fertility of young women, or being used as a method of mass extermination. Awareness of these myths created a desire for trusted information to help guide the parental decision-making process.

*b. Doctors and scientific studies s are trusted sources of COVID-19 information.* Participants expressed a desire for doctors and scientists to provide trustworthy information about the COVID-19 vaccine for themselves and their children. One participant mentioned that “The information that comes from a clinic, investigations by doctors, those are trustworthy.” Specifically, they felt that their primary care doctor and pediatrician played an important role in clarifying existing misinformation and addressing areas of concern and confusion. A participant stated that “I spoke to the pediatrician, who also said that there is clearly bad information about the vaccine because [people] are saying to get the vaccine to lessen symptoms, not that people will not get sick…there’s erroneous information and people become confused.” In this case, clarification of the preventive role of vaccines helped this participant to feel more confident in vaccinating her children.

In addition to seeking information from doctors, participants sought information from scientific studies and sources. Among the mentioned sources were the Centers for Disease Control and Prevention (CDC) and the World Health Organization (WHO). One participant stated that “I believe in the vaccines, and I believe in science and all of that. I think that they’re there for a purpose.” Some participants also mentioned their children’s schools and school districts as trusted sources of science-based information about the COVID-19 vaccination for children. A participant shared that “I as well have gotten information from the district. They gave a lot of information in regard to the vaccines, like how to protect yourself […] There were doctors that came to us and gave us information. I liked it a lot, and through this source, I’ve gotten a lot more information.” The parents’ informational needs were often addressed by obtaining information from these identified trusted sources.

*c. Latinx parents welcome concise and continual information about COVID-19 vaccines* via *mobile phone*. Participants expressed their favorable experiences with the structure and organization of the MVLA intervention as it provided clear and concise information about COVID-19 vaccines. Furthermore, participants described the delivery of information via mobile phone as beneficial to their experience, including the ease of access to the intervention’s information through text messages on their mobile phones. Participants shared that they also liked the structure of the intervention with the material spread out over 4 weeks. This way, they could learn about the COVID-19 vaccines through several interactions with the platform and at their own time and pace.

Participants described the MVLA intervention videos and material as “direct,” “didactic,” “logical,” and “precise.” One participant who completed the intervention noted that they liked the intervention because “it was very didactic, it was not tedious, it didn’t take a lot of time,” and “with just a click you would go into the survey. You would watch videos in a short amount of time.” They also added, “It didn’t take a lot of time to inform yourself.” Another participant expressed gratitude for participating in the intervention, stating, “I would describe the intervention as giving precise and exact information as I see it, and thanks to that, my family was vaccinated.” Several participants shared that their favorite feature of the intervention was that the videos were “clear,” “helpful,” “informative,” and “short” and “provided reliable information.” Most participants expressed a preference for the video format versus the text format as a method of addressing diverse literacy needs. One participant mentioned that “It’s always better to use the videos since many of us have difficulties in reading or other issues.”

#### 3.2.2. Collective Responsibility

*a. Motivations for parents to vaccinate children against COVID-19.* For many participants, the process of deciding whether to vaccinate themselves and their children often involved considering the effects of COVID-19 experienced by close friends and family. One participant stated that “Unfortunately, we did have family members that succumbed to COVID-19…that really made us think, in my home, that we have to take care of ourselves and make the decision to vaccinate.” Protecting those in their home from illness was a driving factor for the participants who chose to vaccinate their children. One participant stated that “I wanted my son to be vaccinated because my husband also became very sick.” Thus, the desire to prevent symptoms already experienced by family members factored into the decision to vaccinate their children. Similarly, participants planned to vaccinate their children to prevent future complications of COVID-19 in vulnerable family members. For example, one participant stated that “I do want to vaccinate [my children] also because, for example, my parents—who are senior citizens—they are already vaccinated, but we don’t want to expose them…” This consideration for both the family’s recent lived experiences and potential future complications resulting from COVID-19 infection was a salient theme during the discussions.

The participants also discussed protecting their children from COVID-19 both at home and at school, especially in the context of children returning to in-person instruction at their schools. In this case, vaccination served to protect family members in the households as well as the children from exposure to COVID-19 at school. A participant stated that “…there has been a lot of [COVID-19] spread, and it’s because of the people who are not vaccinated. And then afterwards the child goes home…” Another stated that “We decided to vaccinate [our children] to have some peace of mind knowing that they were going to be going to school […], It calmed my mind to know that they were safe.” In contemplating whether to vaccinate their children, participants often considered the daily potential exposure to COVID-19 in settings such as schools and its potential impact on family members at home.

#### 3.2.3. Complacency

a. *There is a balance between the perceived risks of COVID-19 and potential vaccine side effects.* Several participants discussed contemplating the risks and benefits of vaccinating their children against COVID-19. The risks included the vaccine’s side effects and potential conflicts with the children’s preexisting health conditions. Some parents expressed needing more time to decide because they doubted the vaccine’s superiority over the body’s natural ability to defend against infectious diseases. One participant stated that “I grew up without so much medication. My children are that way as well. […] Since [the vaccines] are just experiments and the information is not very good or very logical […], I won’t [vaccinate] yet.” Another stated that “Personally, I think that the body can have the same function with or without the vaccine.” Participants related their lack of confidence in the COVID-19 vaccine to their need for contemplation and desire for more information about children’s experiences with the vaccine. Aversion to potential guilt for not vaccinating their children was another salient theme in the parental decision-making process. This guilt was embodied in hypothetical scenarios that they imagined would occur if they chose to not vaccinate, including COVID-19 side effects, complications, and death. One parent expressed of her child that “[If] she gets sick and something even sadder happens, then I’ll feel guilty…I opted to vaccinate her because I’m responsible for her, and the decision to vaccinate her is in my hands”.

#### 3.2.4. Confidence

*a. Adoption of an age and health stratification approach to parental vaccination decision-making.* We identified age stratification or hierarchical ranking of family members by age group or health status as an approach that builds vaccine confidence in Latinx parents. Many participants described using an age-based hierarchy to determine their family’s order of vaccination. For example, some parents vaccinated themselves first, then their teenagers, and finally their younger children and described this approach as a way to gauge vaccine safety and protect all members of the household as opposed to only a few. Parents also alluded to this same approach when vaccinating children with preexisting health conditions, choosing to vaccinate “healthy children” first. A participant stated that “I also decided to vaccinate my children. I have adolescents, and I thought, well, there’s no reason to vaccinate the adolescents if the younger child won’t get vaccinated. And so [we thought] of the future and the wellness of the entire family.” Another stated that “I got it first, so then I could give it to them. Second, I gave it to my daughters, but they don’t have any health problems. Third, I gave it to my son that has many health problems.” Thus, for many families, age and preexisting health conditions factored into parental contemplation of whether to vaccinate their children.

*b. Digital technologies and videos as useful engagement tools.* The use of digital technologies proved to be an effective approach to engaging immigrant families in education about COVID-19 vaccines for children. Participants endorsed the MVLA intervention for mitigating their fear and stress around vaccinating their children against COVID-19. Many participants regarded MVLA as a source of “trusted information” that delivered concise educational materials through their mobile devices. A participant stated that “[MVLA is] a source of information, more than anything giving security to parents, giving us confidence to know more about vaccines.” Several participants said that they benefited from the MVLA intervention as it addressed many of their informational needs as parents. They also described transitioning from feelings of fear regarding vaccinating their children to feeling more confident after participating in the MVLA intervention. A participant shared that “Truthfully, I didn’t want to vaccinate, and neither did my husband. We didn’t want to vaccinate our daughter either, but after listening to the videos, taking information, and seeing there [were] a lot of things that people talked about in the videos—and then we were given information about how it truly was—that’s how I decided to vaccinate myself, my husband, and my daughter. It was very interesting, and it helped a lot.” Topics that the participants found to be helpful included the vaccine’s mechanism and potential side effects as well as other parents’ testimonials about deciding whether to vaccinate themselves and their children. Participants shared that their confidence about vaccinating their children also motivated them to share the information with friends and family. One participant said that “Truthfully, [MVLA] helped me to share with other friends, because we were also very scared because they would say that—the children…many things could happen to them.” Another participant expressed a similar feeling that the digital intervention was useful because it gave them access to information they did not have in their community: “[I] liked that they sent me resources to my telephone saying, watch this short video with information about COVID. We don’t have a lot of information. The more information that we have, the more that we can share with our community.”

In the spring of 2021, when we were conducting focus groups, the Los Angeles Unified School District announced that they would require students to be vaccinated against COVID-19 by January 2022. Several participants expressed that the vaccination mandates created a lot of stress for them. One participant shared that “If you want your child to remain in in-person classes or if you want them to have classes online…That’s a decision that you have to make for your children, and I have my reasons as to why my child was vaccinated, and I have reasons as to why my son returned to school in person, and this is very hard for many […] it’s very controversial. There’s going to be a lot of fights in between parents.” Awareness of conflicting opinions and misinformation regarding COVID-19 vaccination for children created an impetus for further contemplation and seeking more information (see Section 3.2.2). One participant suggested that “I think [schools] should give a conference where maybe a few doctors or people in charge, like pediatricians or scientists […], can clarify a little bit more so that we can feel more secure because [of] the misinformation on social media. And also there’s a lot of panic in the communities.”

Many parents identified stress reduction as an important benefit of participation in MVLA. The participants expressed that the decision to vaccinate their children was more stressful than the decision to vaccinate themselves and that the intervention helped them feel more confident and comfortable in deciding to vaccinate their children against COVID-19 and reduced the stress they previously felt around the decision. One participant shared that “I liked [MVLA] because of all the information that they’re giving. Right now, there’s a lot of people that don’t have good information, and this intervention is giving information so that everyone can become motivated and confident […] about the vaccine against COVID.”

#### 3.2.5. Constraints

*Parental concern about short- and long-term vaccine side effects in children*. A key aspect of the parental decision to not vaccinate their children was concern about the potential for long-term side effects. Parents expressed interest in receiving more information focused on COVID-19 vaccines and children given that most information at the time was centered on adult experiences and side effects. Furthermore, some participants mentioned that obtaining information about vaccine safety and possible side effects from trustworthy sources was a catalyst for risk–benefit discussions with their primary care doctor or pediatrician.

## 4. Discussion

This study provides unique insight into Latinx parental perspectives about COVID-19 vaccination and factors that influence vaccination decision-making. One key finding was that Latinx parents found digital technology to be beneficial in delivering tailored educational information, addressing their specific informational needs as parents, and subsequently increasing their confidence in COVID-19 vaccines. This study contributes novel insights by revealing that Latinx parents experienced notable benefits from utilizing a language-concordant digital intervention to access culturally tailored educational information about COVID-19 vaccines. The inclusion of videos featuring doctors and a community health worker testimonial as trusted sources significantly aided in addressing the diverse health literacy needs of the participants. Interestingly, we observed that vaccinating their children against COVID-19 required more contemplation and information gathering compared to the decision to vaccinate themselves and other adults in the family. Additionally, we also found that Latinx parents felt a sense of collective responsibility to avoid negative health outcomes from COVID-19, and the desire to protect their children at school served as a motivation to vaccinate. However, the study revealed an ongoing need for culturally tailored information about COVID-19 vaccines and boosters, specifically delivered in Spanish and through trusted sources. The utilization of digital technology and videos emerged as a valuable source of continual and incremental information for Latin parents, empowering them to make informed decisions and increasing their confidence in COVID-19 vaccines.

A noteworthy finding was the active information-seeking behavior of participants, as they sought additional information from trustworthy sources to feel more informed about new vaccines, including their side effects, safety, and efficacy in children. This aligns with previous research on COVID-19 vaccination engagement with Latinx communities [20]. Among Black and Latinx communities specifically, studies have shown that individuals prefer a medical professional [14] from a similar racial/ethnic background to endorse the vaccine before they receive it [4,5,8,9]. Parents in our study also raised specific concerns about the long-term effects of vaccination on the health and well-being of their children. These findings are echoed in a study by Ruggiero et al., which found that delaying or declining COVID-19 vaccination was associated with concerns about vaccine side effects and safety among a predominantly non-Hispanic White parent population [21]. Moreover, our study sheds light on the distinct approach parents take when deciding to vaccinate their children compared to the decision to vaccinate themselves as adults. While numerous studies have explored parental beliefs and attitudes toward COVID-19 vaccination, there is a dearth of literature comparing the decision-making processes for children versus adults. The findings from this study highlight the importance of tailoring information for parents to address children-specific concerns.

The study participants provided valuable recommendations for short, clear, and accessible digital information from trusted sources. Leveraging digital technology and mobile phones can effectively disseminate scientific information tailored to the community and from trusted sources through community partnerships. Community organizations have existing ties to the community through established trust and can be powerful partners in providing access to public health information. The COVID-19 pandemic has revealed culture-specific challenges in disseminating important information about vaccines and COVID-19 prevention [5,11,12,35,36]. Furthermore, the rampant dissemination of myths and conspiracy theories via social media is likely to dissuade communities with low levels of digital literacy from the vaccination [4,5,11,35]. In response to these challenges, the MLVA intervention was created to deliver concise accessible and linguistically appropriate vaccine information. An unexpected and significant finding was that even participants with limited English proficiency and lower educational attainment found the mobile phone intervention’s web-based platform and video format to be easily accessible. This finding underscores the importance of utilizing technologies that bridge language and educational barriers, ensuring equitable access to health information and services for marginalized populations.

This study has some limitations. First, we had a relatively small sample size (n = 47). Thus, our study’s findings may have limited generalizability to other high-risk groups or geographic areas. Moreover, the virtual platform used for the focus group sessions likely resulted in self-selection bias against those with limited telephone and internet access. However, by examining parental perspectives and decision-making processes concerning COVID-19 vaccination of children and gathering feedback on their experiences with MVLA, this study provided timely insight that may inform future community outreach efforts. Although most participants expressed satisfaction with the digital intervention, some desired more consistent follow-up communication and increased contact with the MVLA team. The initial intervention design only included monthly follow-up phone calls to participants who were falling behind on the content. However, these calls took place at the end of each month, which left some participants lagging too far behind and unable to complete the intervention. Additionally, several participants would have preferred to have an opportunity to ask questions to the MVLA team. One participant suggested “a space where you can ask questions about the same video, so that someone can do a follow-up in regard to something that wasn’t clear and maybe not have to wait until the next week.” We further recognize some threats to the study’s validity. Though we only recorded audio for translation, the research team included one focus group facilitator and one note-taker to improve descriptive validity. We used open-ended questions in the focus groups, and all members of the research team reached a consensus regarding the salient themes to improve interpretation validity. Lastly, to reduce reactivity bias among participants, the focus groups were led by separate members of the research team who were not involved with the development or implementation of the MVLA intervention. However, despite these efforts, we acknowledge the threats to validity and researcher bias inherent to qualitative research.

Our findings suggest that parentally engaging vaccine outreach should expand parents’ access to primary care doctors as trusted sources of information and tailor information to address the COVID-19 vaccine’s potential long-term side effects and ability to keep children who return to school—and their families—safe. As the participants contemplated vaccinating their children, they considered their own confidence in the COVID-19 vaccine’s ability to prevent their children from contracting and spreading the virus within their households and families. The Latinx parents expressed that their need for more information tailored to children in their communities was critical to their decision to vaccinate their young children against COVID-19. Specifically, they needed information from trustworthy sources, including doctors and other health care professionals, scientific studies, the CDC, the WHO, and their school districts. These sources of information were a key component of the participant’s ability to discredit misinformation, myths, and conspiracy theories. Participants also cited MVLA as a source of reliable information as it contained many of the features and information streams listed above. Therefore, it is important to foster and expand community-based outreach efforts to include trusted messengers ranging from community leaders to scientists, physicians, and officials. These sources can be further incorporated into future iterations of MVLA or other, similar efforts. Future public health efforts for community members with limited English proficiency in under-resourced communities should focus on delivering timely, accessible, and up-to-date information about COVID-19 vaccines, boosters, and prevention strategies and resources on how to access vaccines using innovative, mobile-based approaches. Finally, public health outreach should address culturally specific myths and barriers to vaccination in the Latinx community.

## 5. Conclusions

In conclusion, the focus group study presented above has significant practice, research, policy, and public health implications. The findings highlight the importance of culturally tailored and language-concordant interventions. Specifically, this study resulted in the design of an improved version of the intervention, MVLA 2.0, to meet the informational needs and preferences of Latinx parents. Future interventions can consider providing clear and concise information about COVID-19 vaccines through digital technology such as mobile phones and can utilize video format to accommodate diverse literacy needs. Furthermore, the study emphasizes the role of doctors and pediatricians as trusted sources of information and the need for effective doctor–patient communication. From a research perspective, this study contributes to a deeper understanding of Latinx parental perspectives and decision-making factors related to COVID-19 vaccination for their children. The study reveals parents’ concerns about the potential long-term side effects of COVID-19 vaccines in children. Future research can focus on investigating and providing evidence on the long-term safety and efficacy of COVID-19 vaccines specifically in pediatric populations. The study acknowledges the stress and controversy surrounding vaccination mandates in schools. Policymakers should consider the concerns and information needs of parents when implementing vaccination mandates and communicate effectively with parents, providing clear and reliable information about the benefits and safety of COVID-19 vaccines for children. Public health efforts should prioritize the dissemination of accurate and reliable information about COVID-19 vaccines through trusted sources, such as doctors, scientific studies, and reputable organizations such as the CDC and WHO. Efforts should be made to counteract misinformation circulating within social networks. Overall, these insights can guide the development of interventions, policies, and public health strategies to promote vaccine confidence and health equity in the Latinx community.

## Figures and Tables

**Table 1:** Sample qualitative questions.

Selected Interview Questions *
‣ What have you or members of your community heard about vaccines to protect against COVID-19 in children ages 5–11 years? ‣ We would like to get to know your perspectives and approach to making decisions regarding vaccines for your children. In general, what considerations do you make when taking your child for COVID-19 vaccination? ‣ What additional information do you need to feel comfortable receiving the COVID-19 vaccine for your child? ‣ Where would you feel most comfortable getting the vaccine? ‣ If you were to describe MyShotLA to a friend, how would you describe it?

* Full guide available upon request.

**Table 2:** Characteristics of focus group participants.

Participant Demographics (N = 47)	No. (%)
**Age of parent (mean, SD)**	41.3 (7.9)
**Preferred language**	
Spanish	42 (89.4)
**Mean number of minors in household**	2.3 (1.2)
**Parent COVID-19 vaccination status**	
Vaccinated	35 (74.5)
Not vaccinated	12 (25.5)
**Ethnicity**	
Mexican/Mexican American/Chicano	31 (-)
Other Hispanic/Latino/Spanish Origin	16 (-)
**Born outside of the U.S.**	38 (80.9)
**Highest education level attained**	
Some high school or less	21 (44.7)
High school graduate/GED	17 (36.2)
Some college or more	9 (19.2)
**Employment status**	
Employed	12 (25.5)
Unemployed	11 (23.4)
Other	24 (-)
**Household income (US dollars)**	
<$25,000	38 (80.9)
$25,000–$49,000	6 (12.8)
≥$50,000	2 (4.3)
Prefer not to respond	1 (2.1)
**Health insurance status**	
Insured	27 (57.5)
Not insured	12 (25.5)
Do not know/Prefer not to respond	8 (17.0)
**At least one minor in household aged 2–11 years**	
Yes	38 (80.9)

**Table 3:** Main themes and subthemes for building vaccine confidence using the 5Cs categories.

5Cs Categories	Themes/Subthemes
Calculation (preference for deliberation)	‣ Vaccinating children against COVID-19 requires more contemplation than vaccinating adults ‣ Doctors and scientific studies are trusted sources of COVID-19 vaccine information ‣ Latinx parents appreciate continual and incremental information about COVID-19 vaccines via mobile phone
Collective responsibility (communal orientation)	‣ Motivations for parents to vaccinate children against COVID-19 include: ○ Previous experiences with COVID-19 ○ Desire to protect children at school
Complacency (perceived personal health status and invulnerability)	‣ There is a balance between perceived risks of COVID-19 and potential vaccine side effects
Confidence (attitude)	‣ Adoption of an age and health stratification approach to vaccination ‣ Digital technologies and videos as useful engagement tools
Constraints (self-control)	‣ Parental concern about short- and long-term effects of the vaccine in children

## Data Availability

The data presented in this study are available on request from the corresponding author and upon IRB approval. The data are not publicly available due to IRB restrictions.
